# Giant congenital melanocytic nevus in a Cameroonian child: a case report

**DOI:** 10.1186/s13256-018-1707-y

**Published:** 2018-06-23

**Authors:** Francky Teddy Endomba, Charlie Romain Mbega, Joel Noutakdie Tochie, Saint-Just N. Petnga

**Affiliations:** 1 Department of Internal Medicine and Sub-specialties, Faculty of Medicine and Biomedical Sciences, University of Yaoundé I, Yaoundé, Cameroon; 2 Department of Anesthesiology and Intensive Care Medicine, Faculty of Medicine and Biomedical Sciences, University of Yaoundé I, Yaoundé, Cameroon; 3 Health and Human Development (2HD) Research Group, Douala, Littoral Region Cameroon; 4 Specialized Internship Program, Faculty of Medicine and Biomedical Sciences, University of Yaoundé I, P.O. Box 1364, Yaoundé, Cameroon

**Keywords:** Giant congenital melanocytic melanoma, “Turtle child”, Cameroon

## Abstract

**Background:**

Giant congenital melanocytic nevus is a very rare condition characterized by a large skin lesion and an increased risk of complications like neurocutaneous melanosis and malignant transformation. Reports of giant congenital melanocytic nevus are scarce in the sub-Saharan African literature and here we present a case of this disease in a Cameroonian adolescent.

**Case presentation:**

A 12-year-old Cameroonian girl from the "Baka" ethnic group, with no relevant family and medical histories presented with a progressively extensive brownish-black nodular hypertrophic skin lesion of approximately 45 cm, which she had had since she was 2-days old. The lesion covered her entire back giving an appearance of “turtle child”, which was highly suggestive of a giant congenital melanocytic nevus. She was booked in for a surgical intervention organized by a health campaign within her community. Meanwhile she was provided with psychological support and her family was counseled on warning signs of complications which would warrant an urgent consultation.

**Conclusions:**

Here we presented a case of giant congenital melanocytic nevus, apparently the first in the Cameroonian literature. In view of the potential severe complications as well as psychological trauma of this pathology, we draw clinicians’ attention to this extremely rare but real pathology in our country, for a timely diagnosis and management.

## Background

Melanocytic nevi refer to tumor-like malformations of the skin or mucous membrane, due to benign proliferation of pigment-producing skin cells called melanocytes [1]. They belong to the spectrum of melanocytic neoplasms which includes malignant varieties such as melanoma. Based on their onset, melanocytic nevi are usually classified into either congenital or acquired forms [1, 2]. Congenital nevi developed from neuro-ectodermal cells and are further classified depending on the diameter of the nevus; a nevus is classified as a giant congenital melanocytic nevus (GCMN) when the diameter of the largest nevus exceeds 20 cm [3]. Although congenital melanocytic nevi (CMN) are relatively common, GCMN is a rare entity with an incidence of 1 in 20,000 to 500,000 live births [4]. Its diagnosis is mainly clinical, but may be reinforced by the histological findings. In addition to its rarity, GCMN is of interest because of its potentially severe complications, namely melanoma and neurocutaneous melanosis (NCM), due to central nervous system involvement [1, 2, 5]. Furthermore, there is considerable psychological trauma associated with the disfiguring lesions of GCMN, particularly when it affects females [4–6]. GCMN has been less reported in sub-Saharan Africa. Here we report a case of GCMN in a Cameroonian girl in a bid to draw the attention of clinicians to this pathology.

## Case presentation

**Fig. 1:**
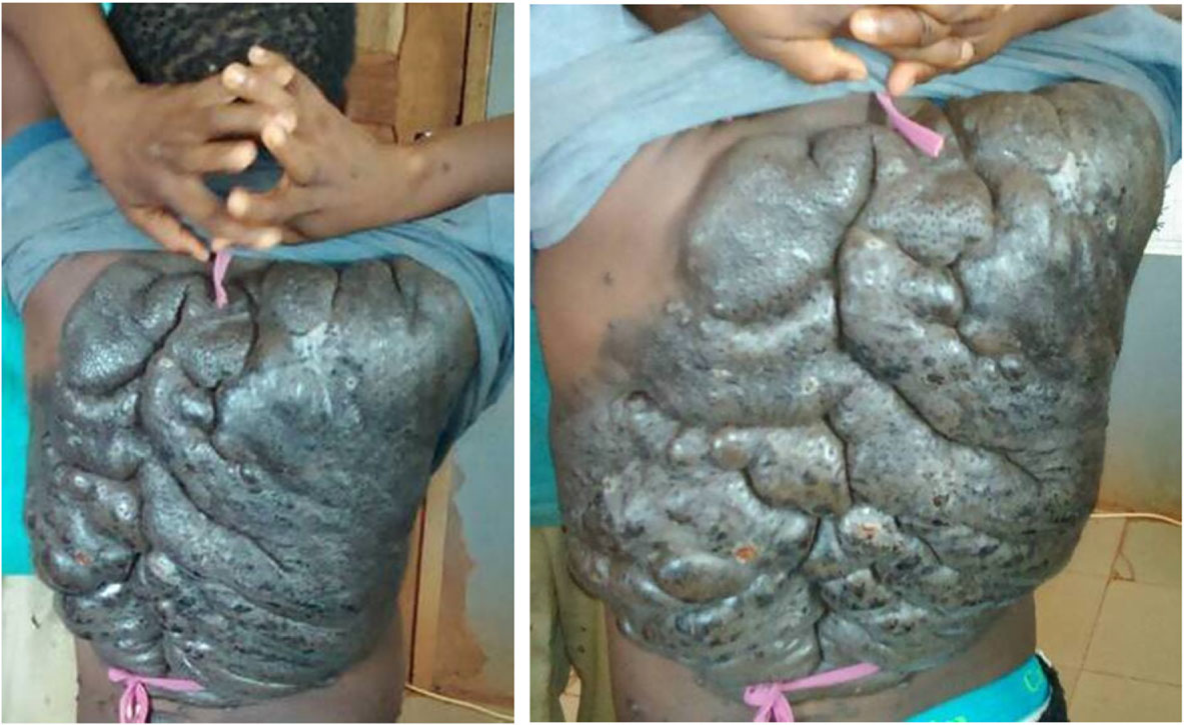
Hypertrophic nodular brownish-black lesions of the back suggestive of a giant congenital melanocytic nevus

A 12-year-old Cameroonian girl from the "Baka" ethnic group and residing in a remote area of the East region of Cameroon presented with a progressively extensive, pruritic, and painless pigmented skin lesion on her back, persistent since she was 2-days old. She was born through normal vaginal delivery at term from an uneventful pregnancy. Her past medical and family histories were unremarkable. On our initial physical examination, she had normal anthropometric characteristics for age, as well as normal vital parameters. Examination of her skin revealed a large, irregular, well-demarcated and unequally pigmented (bluish-brown to black) multinodular hypertrophic nevus occupying almost all her back (Fig. 1). The largest diameter of this lesion was 45 cm. Its surface was rough and had several excoriation marks. No other malformation was apparent. An examination of her lungs, heart, abdomen, and extremities was otherwise normal. Despite the unavailability of histopathology in our setting, the aforementioned clinical findings were highly suggestive of a GCMN. She was scheduled for a free of charge surgical campaign due within the same year in her community. This surgical excision would provide several benefits namely the reduction of the risk of melanoma, improvement in aesthetics, and obtaining histopathology samples. Taking into consideration the psychosocial aspect of this pathology, our patient and her parents were also oriented to the consult of a psychologist. Meanwhile, her parents were counseled on signs of complications which should warrant urgent admission. At 3-month follow-up, she was still pending surgical intervention. Currently, she is being followed-up clinically and psychologically on a weekly basis while waiting for surgery.

## Discussion

CMN are pigmented skin lesions formed by epidermal and dermal-derived nevi cells (also known as melanocytic cells), which can be present at birth or may develop after a few weeks of life [1, 2, 4, 7]. CMN differs from the acquired form of melanocytic nevus by its presence at birth, tendency to increase in size, and the risk of malignant transformation [7, 8]. As previously mentioned, CMN has been classified by many authors based on the size of the lesions. This stems from the fact that the risk of complications is proportional to the maximum diameter of the nevus [3, 4]. Of all the classifications of CMN put forth, the most universally accepted is that described by Kopf *et al.* [3]. Kopf and colleagues [3] proposed three distinct types of CMN based on the largest diameter of the nevus: small (< 1.5 cm), medium (from 1.5 to 19.9 cm), and lastly, large or GCMN (≥ 20 cm) to which our patient belonged.

With respect to epidemiology, approximately 1% of live births presents with a CMN. The small form occurs in 1 per 100 live births, the medium form in 6 per 1000 live births, and the incidence of the giant form is globally estimated at 1 in 20,000 to 500,000 live births [4, 9]. In general, a preponderance of individuals with CMN is female as exemplified in our case. The reported female-to-male ratio ranges from 1.17:1 to 1.46:1 [4, 9]. Of note, CMN occur mostly in a sporadic pattern with very few reports of familial predisposition as observed in our patient. Genetic mutations are the basis of the pathogenesis. It has therefore been demonstrated that CMN, especially its medium and giant (94.7%) forms, frequently harbor *NRAS* mutations and to a lesser extent *BRAF* mutations [10, 11]. The *NRAS* gene, located on the short arm of chromosome one at position 13.2, provides instructions for making a protein called N-Ras that is involved primarily in regulating cell division [11]. A mutation of this gene thus leads to a morphological error in the neuro-ectoderm during the fourth and sixth weeks of gestation with uncontrolled growth of melanocytes precursor cells known as melanoblasts, leading to the formation of CMN [4, 7]. Furthermore, there is also a molecular component in this pathogenesis, involving the hepatocyte growth factor or scatter factor (HGF/SF), a cytokine partially associated with the control of the development of melanocytes [4, 8].

Classically, GCMN is an asymptomatic disease but some patients may complain of pruritus and xerosis as shown in the case above. The findings on physical examination are variable but affected persons usually present with brownish to black, flat or nodular, well-defined lesions, associated with hypertrichosis. The surface of the nevus may be papular, warty, cerebriform, or rough, as in our case [1, 2, 4, 11]. GCMN can affect any region of the skin but it is mostly located on the trunk. Some specific locations lead to typical clinical presentations which are highly suggestive of GCMN. For instance, “bathing trunk” when the lesion is located in the sacral and perineal areas, and “turtle child” when there is involvement of all the back, as seen in the index case [4]. Some smaller pigmented lesions scattered over the skin surface and called satellite lesions can be found in up to 78% of cases [4].

The repercussion of GCMN can be organic or not. In fact, it is well established that the disfiguring character of this pathology may have psychosocial implications with deleterious impacts on the self-esteem of affected individuals, especially females [4–6]; this psychological trauma may culminate in social exclusion as was nearly the case in our patient. The most serious organic complications are NCM and the evolution to cutaneous melanoma (1–5% of cases) [7]. It should be noted that GCMN greater than 40 or 50 cm in diameter and having more than 20 satellite lesions predispose to those severe conditions [4, 9, 12]. Although not observed in our case, some congenital anomalies are often concomitantly associated with GCMN and they may worsen the prognosis of the GCMN: spina bifida occulta, meningocele, club foot, neurofibromatosis, lipomatosis, and hypertrophy and atrophy of limbs [13].

Although the diagnosis of GCMN is mainly clinical, histopathology study when available is needed to affirm the diagnosis and rule out malignant transformations. In this regard, the most common histological findings are hyperkeratosis and hyperplasia, elongation of epidermal ridges, and increased number of melanocytes [4].

Finally, the therapeutic options for GCMN vary from surgical excision to laser therapy. Treatment should be individualized, taking into consideration the age of the patient, the size and location of the lesions, the risk of melanoma, the possibility of a NCM, and the presence of other congenital abnormalities or comorbidities. The prognosis depends on all of these factors [4, 14, 15].

## Conclusions

Here we presented the case of GCMN in a Cameroonian girl, apparently the first in the Cameroonian literature. Although a very rare pathology, its diagnosis is mainly clinical and has a pivotal role in its timely management geared at averting its associated psychological trauma and risk of complications such as NCM and malignancies. We therefore draw clinicians’ attention to this extremely rare but real pathology in our country, in order to ameliorate its prognosis.

## Abbreviations

CMN: Congenital melanocytic nevi
GCMN: Giant congenital melanocytic nevus
HGF: Hepatocyte growth factor
NCM: Neurocutaneous melanosis
SF: Scatter factor
